# A routine intervention in a highly unusual vessel

**DOI:** 10.1007/s12471-021-01635-x

**Published:** 2021-09-15

**Authors:** A. Gasecka, M. Voskuil, E. E. C. de Waal, M. I. F. J. Oerlemans, F. Ramjankhan, L. W. van Laake, A. O. Kraaijeveld

**Affiliations:** 1 Department of Cardiology, University Medical Centre Utrecht, Utrecht, The Netherlands; 2 Department of Anaesthesiology, University Medical Centre Utrecht, Utrecht, The Netherlands; 3 Department of Cardiothoracic Surgery, University Medical Centre Utrecht, Utrecht, The Netherlands

A 31-year-old woman with a HeartWare^TM^ (Medtronic, Minneapolis, MN, USA) left ventricular assist device (LVAD), which had been implanted for ischaemic cardiomyopathy, presented with progressive dyspnoea. Bilateral pneumonia was suspected. Despite antibiotic treatment, the patient’s condition deteriorated: she became more dyspnoeic and developed cardiogenic shock with a low LVAD flow. Computed tomography angiography showed an intraluminal focal outflow graft stenosis. In a multidisciplinary team discussion, the patient was scheduled for an emergency percutaneous intervention via a femoral approach to avoid surgery, based on previous reports [1–3]. Angiography confirmed the stenosis, with an invasive peak-peak gradient of 80 mm Hg (Fig. 1a). This was treated with an Advanta V12 balloon-expandable covered stent (10 mm × 38 mm) and post-dilated with an Advance balloon (10 mm × 20 mm), resulting in a residual gradient of 10 mm Hg and an immediate increase in LVAD flow (Fig. 1b). The patient recovered uneventfully after this procedure.

**Fig. 1:**
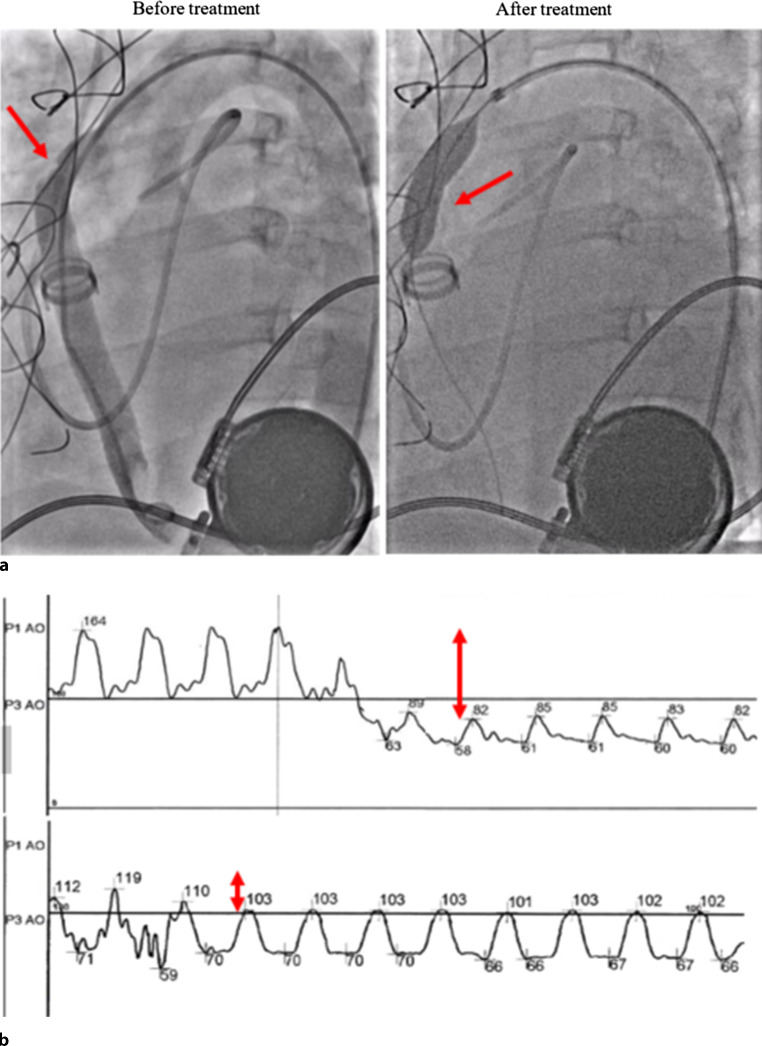
**a** Angiography confirming outflow graft stenosis (*left panel*, *red arrow*). Advanta V12 balloon-expandable covered stent (10 mm × 38 mm) in outflow tract (*right panel*, *red arrow*). **b** Invasive blood pressure monitoring demonstrating an 80-mm Hg gradient over the stenosis before the procedure (*upper panel*, *red arrows*) and a residual 10-mm Hg gradient after the procedure (*lower panel*, *red arrows*)

The incidence of outflow graft stenosis ranges from 0.01 to 0.03 per patient-year [4, 5]. Personalised anticoagulation protocols and surgical implantation techniques are currently being studied to prevent LVAD outflow graft obstruction due to stenosis, thrombosis or torsion.

## References

[1] Wert L, Kaufmann F, Solowjowa N (2021). Diagnosis and treatment strategies of outflow graft obstruction in the fully magnetically levitated continuous-flow centrifugal left ventricular assist device: a multicenter case series. ASAIO J.

[2] Gertz ZM, Trankle CR, Grizzard JD (2021). An interventional approach to left ventricular assist device outflow graft obstruction. Catheter Cardiovasc Interv.

[3] Nathan S, Ghotra AS, Rajagopal K (2020). Left ventricular assist device outflow graft obstruction: a case series. ASAIO J.

[4] Burke MA, Alexy T, Kamioka N (2020). Outflow graft obstruction causing recurrent heart failure after left ventricular assist device implantation. J Heart Lung Transplant.

[5] Scandroglio AM, Kaufmann F, Pieri M (2016). Diagnosis and treatment algorithm for blood flow obstructions in patients with left ventricular assist device. J Am Coll Cardiol.

